# Blood–Brain Barrier Dysfunction and Aβ42/40 Ratio Dose-Dependent Modulation with the ApoE Genotype within the ATN Framework

**DOI:** 10.3390/ijms241512151

**Published:** 2023-07-29

**Authors:** Sofia Toniolo, Francesco Di Lorenzo, Sergio Bernardini, Nicola Biagio Mercuri, Giulia Maria Sancesario

**Affiliations:** 1 Cognitive Neurology Group, Nuffield Department of Clinical Neurosciences, University of Oxford, Oxford OX1 3AZ, UK; 2 Department of Systems Medicine, University of Rome ‘Tor Vergata’, 00133 Rome, Italyg.sancesario@hsantalucia.it (G.M.S.); 3 Non-Invasive Brain Simulation Unit, IRCSS Santa Lucia Foundation, 00179 Rome, Italy; 4 Biobank Unit, IRCSS Santa Lucia Foundation, 00179 Rome, Italy

**Keywords:** ATN, blood–brain barrier, Alzheimer’s disease, Aβ amyloid, ApoE

## Abstract

The definition of Alzheimer’s disease (AD) now considers the presence of the markers of amyloid (A), tau deposition (T), and neurodegeneration (N) essential for diagnosis. AD patients have been reported to have increased blood–brain barrier (BBB) dysfunction, but that has not been tested within the ATN framework so far. As the field is moving towards the use of blood-based biomarkers, the relationship between BBB disruption and AD-specific biomarkers requires considerable attention. Moreover, other factors have been previously implicated in modulating BBB permeability, including age, gender, and ApoE status. A total of 172 cognitively impaired individuals underwent cerebrospinal fluid (CSF) analysis for AD biomarkers, and data on BBB dysfunction, demographics, and ApoE status were collected. Our data showed that there was no difference in BBB dysfunction across different ATN subtypes, and that BBB damage was not correlated with cognitive impairment. However, patients with BBB disruption, if measured with a high Qalb, had low Aβ40 levels. ApoE status did not affect BBB function but had a dose-dependent effect on the Aβ42/40 ratio. These results might highlight the importance of understanding dynamic changes across the BBB in future studies in patients with AD.

## 1. Introduction

The definition of Alzheimer’s disease (AD) has undergone a progressive shift from a clinical to a biological construct, with biomarkers of amyloid status and tau pathology now being essential for in vivo diagnosis [1]. Indeed, the prototypical multidomain amnestic dementia phenotype, which has historically been used to define AD [2], does not necessarily imply the presence of AD pathologic change at autopsy [3,4]. This clinical phenotype could be underpinned by other neuropathological entities, such as Limbic-predominant age-related TDP-43 encephalopathy (LATE) [5], primary age-related tauopathy (PART) [6], α-synucleinopathy [7], and argyrophilic grain disease [8]. The presence of mixed pathology at autopsy is extremely high, especially in older patients [9]. Furthermore, non-amnestic clinical presentations, with an early impairment of language, visuospatial, and executive functions, have been embedded into the biological definition of AD [1]. Therefore, relying on cerebrospinal fluid (CSF) biomarkers in the diagnosis of AD seems pivotal to identify in vivo evidence of AD-specific neuropathology. This has exerted a huge impact on the perception of CSF analysis in neurodegenerative diseases, transforming its application from a test with negative predictive value in ruling out inflammatory or infectious diseases to a positive predictive value in diagnosing AD.

While many different CSF biomarker ratios have been evaluated to increase diagnostic accuracy [10], since the introduction of the amyloid (A), tau (T), and neurodegeneration (N) ‘ATN’ system, each single biomarker is regarded as reflecting a separate construct, the combination of which will determine diagnostic labelling [1]. In the context of CSF biomarkers, phosphorylated tau (p-tau) reflects the tau (T) status of an individual, the total presence (t-tau) of neurodegeneration (N), and either amyloid β42 (Aβ42) or the Aβ42/40 ratio of the pathological amyloid deposition (A) in the brain. The Aβ42/40 ratio outperforms Aβ42 alone in increasing diagnostic accuracy, given the wide range of interindividual variability in the rate of production and drainage of amyloid [11]. There is no consensus whether Aβ40 levels remain substantially unaffected in the various forms of dementia [12,13,14], or even show higher values in patients with AD [15,16], and mild cognitive impairment (MCI) [11]. However, the selective decrease in Aβ42 compared to constant or even elevated Aβ40 seems to be specific for AD, with a cut-off of 0.6 for the Aβ42/40 ratio showing good diagnostic accuracy [15], and a stronger correlation than Aβ42 alone to amyloid positron emission tomography (PET) [17] and AD neuropathology [18].

Although Aβ is found in the blood at the same concentration of CSF (0.1–0.5 nM), Aβ deposits are largely found in the interstitial compartments within the brain [19]. The relationship between serum and CSF levels of Aβ and tau peptides has been the target of the recent diagnostic efforts to develop blood-based biomarkers, which are now being extensively validated and increasingly incorporated into the design of clinical trial [20,21,22,23,24]. In this era of intensive shift to blood-based assays, it is crucial to investigate the fine-grain balance between the serum and CSF concentration of AD-specific biomarkers, such as t-tau, p-tau, Aβ42, Aβ40, and the Aβ42/40 ratio, but also to understand if their relationship with other molecules such as albumin may indirectly play a role.

The role of the alteration of the blood–brain barrier (BBB) in AD patients has been widely investigate [25]. The accumulation of Aβ in the vascular wall may lead to endothelial cell damage and cause BBB disruption in AD patients [26]. A recent metanalysis showed an increased BBB dysfunction in patients with AD compared to controls, using either the CSF/serum albumin index (Qalb) or the IgG index [27]. Some evidence suggests that only a very small percentage of patients with AD show increased intrathecal IgG synthesis, while approximately 25–40% of patients could show dysfunction of the BBB if measured with Qalb [28,29,30].

Previous studies found that AD cases with evidence of concomitant cerebrovascular pathology have high Qalb as a sign of impaired BBB function, and Qalb correlated positively with CSF biomarkers of angiogenesis and endothelial dysfunction rather than AD-specific biomarkers [29,31], while other studies showed no correlation with vascular comorbidities [32,33]. Age and gender have been associated with increased BBB permeability, with elderly patients showing higher levels of BBB dysfunction [34] and recent evidence suggesting potentially higher Qalb values in men [35]. Moreover, Qalb shows an increased variability in individuals over 45 years of age, suggesting not only an impairment, but also a higher variability in elderly subjects, suggesting a less stable BBB compared to younger individuals [36]. Previous evidence found that BBB breakdown may be linked to Apolipoprotein E 4 allele (ApoE4), the major genetic risk factor for late-onset AD [37,38], while some studies failed to find such an association [32,39].

Moreover, there is conflicting evidence of an association between Qalb and cognitive impairment for standard neuropsychological measures such as the MMSE (Mini Mental State Examination) or the MoCA (Montreal Cognitive Assessment), and between Qalb and annual change on the MMSE and CDR (Clinical Dementia Rating Scale) [32]. Interestingly, Qalb changes do not seem to be related to clinical progression or change in the transition between preclinical and prodromal AD [39]. Lastly, a dysfunctional BBB is not deemed to be exclusively associated with AD, but may be found in many neurodegenerative diseases, as well as in patients with no neurological diseases [40].

The aim of our study was to investigate the relationship between biomarkers of BBB damage and AD-specific CSF biomarkers. We assessed the relationship between BBB biomarkers (total protein, CSF and serum albumin, the CSF/serum albumin ratio (Qalb), serum and CSF IgG, and the IgG index), CSF markers of AD (Aβ42, Aβ40, Aβ42/40 ratio, p-tau, and t-tau), age, sex, ApoE status, and cognitive status. Moreover, we compared biomarkers of BBB disruption between groups characterised using the ATN biomarker framework.

## 2. Results

### 2.1. Overview of Participants within the ATN Framework and Between-Group Comparisons

According to the ATN criteria, 57 patients showed a normal biomarker profile (A-T-N-), 21 met the criteria for Alzheimer’s pathologic change (A+T-N-), 70 were classified as Alzheimer’s disease (A+T+N-, A+T+N+), and 24 as non-Alzheimer’s pathologic change (A-T+N-, A-T+N+) [1], Table 1. Only one patient met the criteria for Alzheimer’s and concomitant non-Alzheimer’s pathologic change (A+T-N+) and was therefore excluded from subsequent analysis.

Groups were not statistically different in age, sex, MMSE scores, or ApoE status, as shown in Table 1. As expected, the four groups differed in terms of CSF total-tau, p-tau, Aβ42, Aβ40, and the Aβ42/40 ratio (post-hoc analysis in Supplementary Materials Table S1).

We found no difference in any of the BBB biomarkers (protein, serum albumin, CSF albumin, Qalb, serum IgG, CSF IgG, IgG index) among the four groups (Table 2), nor among any group pair after post-hoc Bonferroni correction. Only 1.16% of the patients had abnormal IgG index (>0.7), while 31.2% of our sample had abnormal Qalb, and the percentage was not different among groups.

### 2.2. Correlation between BBB and AD-Specific Biomarkers

**Figure 1:**
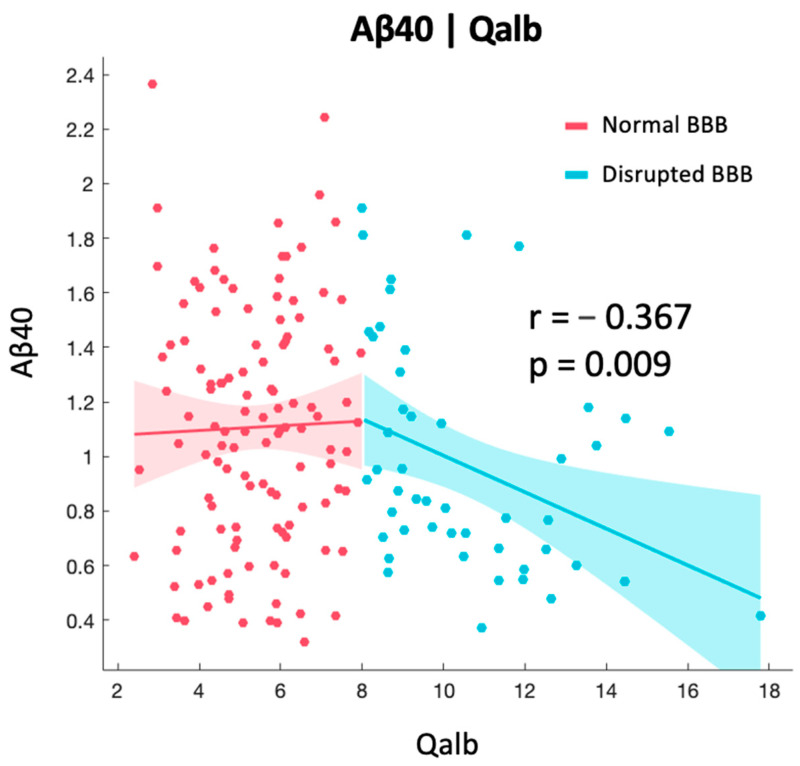
Correlations between Qalb and Aβ40 split by integrity of BBB. Aβ40 concentrations expressed as pg/mL.

Qalb was correlated with Aβ40 levels (r = −0.190, *p* = 0.014), but not with Aβ42 or the Aβ42/40 ratio. Patients were then divided according to the integrity of their BBB using the standard age-related Qalb cut-off of eight as having normal Qalb (<8) or high Qalb (>8). We found that the negative correlation between Aβ40 and Qalb was only significant (r = −0.367, *p* = 0.009) in patients with high Qalb, while in patients with normal Qalb this was not observed (Figure 1). No other significant correlations were found.

### 2.3. Relationship between BBB Biomarkers, Demographics, and Cognition

**Figure 2:**
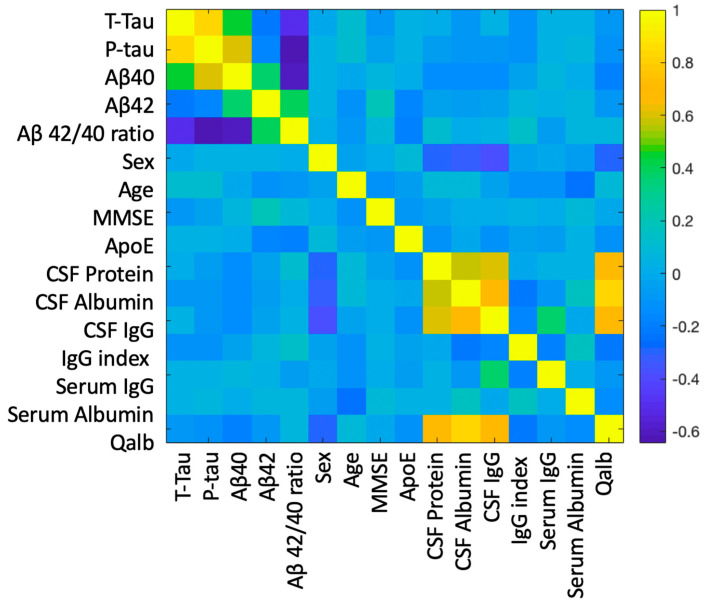
Correlation matrix across all variables. R values are represented by graded colour, with negative correlations in blue and positive correlations in yellow.

MMSE scores were not correlated with the BBB markers studied but were positively correlated with Aβ42 levels, with lower MMSE associated with lower levels of Aβ42 (r = 0.227, *p* = 0.011). Age was negatively correlated with serum albumin (r = −0.240, *p* = 0.001), with lower levels of serum albumin seen in elderly individuals. Females had lower CSF proteins (W = 5084, *p* < 0.001), CSF albumin (W = 4825, *p* < 0.001), CSF IgG (W = 4951, *p* < 0.001), and Qalb (W = 4942, *p* < 0.001). A correlation matrix across all variables in shown in Figure 2.

**Figure 3:**
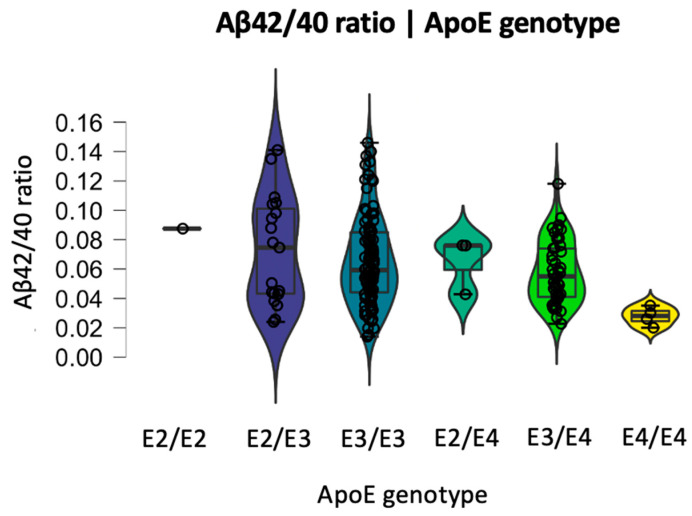
Aβ42/40 ratio dose-dependent modulation by APOE genotype.

The Kruskal–Wallis group comparison among different ApoE groups divided by graded risk factor showed that carrying a more high-risk genetic profile did not affect BBB biomarkers but was associated with lower levels of Aβ42/40 ratio in a dose-dependent manner (H (4) = 9.973, *p* = 0.041), as seen in Figure 3. As there was only one subject with E2/E2 genotype, that subject was excluded from statistical analysis but kept for data visualization purposes.

### 2.4. Principal Component Analysis

**Figure 4:**
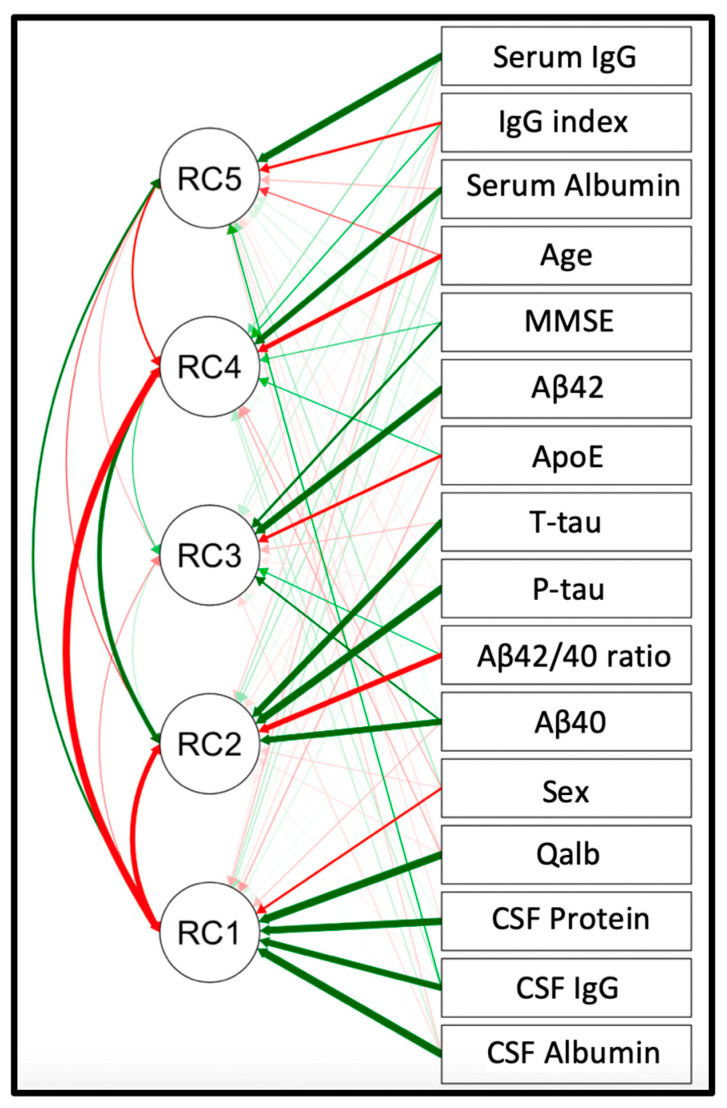
PCA path diagram.

A PCA was then performed to reduce the dimensionality of our dataset and reveal the latent structure of the relationship between the different factors in our data. A five-factor model was able to reflect the data efficiently (PCA model value = 3783, df = 50, *p* < 0.001), see Figure 4 and Table 3 and Table 4.

The first component (RC1) could reflect sex-related differences in CSF protein (total protein, albumin, IgG) content and Qalb, with males showing higher levels of BBB disruption. The second component (RC2) reflects AD-specific CSF biomarkers, with higher t-tau and p-tau correlating negatively with Aβ42/40 ratio, as expected. The third component (RC3) might reflect the complex interplay between Aβ species, ApoE status, and cognition. The fourth component (RC4) seems to mirror the well-known age effect on serum albumin, with older age being associated with lower serum albumin. The fifth component (RC5) could represent a systemic inflammatory response. To note, RC1, RC2, and RC4 are highly correlated, as seen in Figure 4 and Table 4.

## 3. Discussion

In light of the recent advent of biomarkers to measure ATN status in blood, a deeper understanding of the impact of BBB dysfunction on AD biomarkers is warranted. Beyond its importance in determining the fine-grain balance between the concentration of AD-specific biomarkers in the blood and in the CSF for diagnostic purposes, this might also extend to a better evaluation of success rates of therapeutic interventions if a future shift to blood-based biomarkers is implemented as a surrogate for CSF and PET imaging to determine clinical trial endpoints. We found no difference in any of the BBB biomarkers (protein, serum albumin, CSF albumin, Qalb, serum IgG, CSF IgG, IgG index) among the four groups divided by their ATN profile, thus confirming previous data based on the clinical stratification of patients [39]. In line with previous evidence, only a small percentage of patients (1.16%) had abnormal IgG index (>0.7), while 31.2% of our sample had abnormal Qalb, and that was independent from their ATN profile. Therefore, BBB can be altered in a substantial proportion of patients, but it is not a unique feature of AD, as could be present in many other neurological conditions, as previously reported [39,40].

Our data also showed that only when the BBB is damaged, if measured by a high Qalb, Aβ40 levels decrease. Albumin is the most abundant protein in blood, with a concentration of 640 μM, but has a markedly reduced concentration in the CSF of typically 3 μM [41]. At CSF concentrations, albumin inhibits the kinetics of Aβ fibrillization, significantly increasing the lag time and decreasing the total amount of fibrils produced, with the amount of amyloid fibres generated directly correlating to the proportion of Aβ not competitively bound to albumin [42]. In the absence of CSF albumin, typical fibre morphology is observed with numerous fibres generated, while in the presence of albumin at 5–50 μM levels, almost no fibres or oligomers of either Aβ40 and Aβ42 are detectable [42]. This suggests that albumin binds to Aβ molecules and traps them in a non-fibrillar form so that they are not available to form fibres. Moreover, we know that Aβ40 monomers inhibit the aggregation of non-toxic Aβ42 monomers, in an Aβ42/40-ratio-dependent manner [43]. Aβ40 can also release Aβ42 monomers from Aβ42 aggregates, thus exhibiting a protective role by competing with Aβ42 monomers [43]. Based on these findings, new therapeutic approaches have emerged, such as plasma exchange, where albumin replacement in the serum is thought to induce the shifting of the dynamic equilibrium existing between brain and plasma Aβ [44]. The underlying hypothesis is that plasma exchange-mediated sequestration of albumin-bound Aβ in plasma would increase the transport of free Aβ from CSF to plasma, thereby decreasing brain Aβ burden [44]. Preliminary results of the Alzheimer’s management by albumin replacement (AMBAR) study show improvements in short-term verbal memory, language fluency, and processing speed in mild–moderate AD patients who underwent plasma exchange [45], but more evidence is needed to assess its replicability and the feasibility of large-scale implementation. Our data highlight the fact that BBB damage did not differ between patients within the ATN framework, which might be important for patient selection in future clinical trials. However, BBB disruption was associated with lower Aβ40 levels. As Aβ40 has been proposed as a protective factor, being able to dynamically compete with Aβ42 in fibril formation, one might postulate that BBB disruption might lead to lower Aβ40 levels being available to compete with Aβ42 and being potentially detrimental. However, whether this represents a positive or a maladaptive response needs further investigation.

BBB biomarkers were not impacted by the ApoE genotype, but our data support a modulation of the Aβ42/40 ratio with ApoE status. This seems to follow a dose-dependent effect, with ApoE phenotypes at higher risk of developing AD being associated with a lower Aβ42/40 ratio. This result is in line with the previous literature on the association between carrying at least one ApoE4 allele and lower levels of amyloid but not higher levels of tau in the CSF [46], but extends these findings to participants with different ATN statuses. Moreover, it shows a dose-dependent effect across different genetic subtypes including participants with at least one ApoE2 allele. We were also able to show that ApoE status was not associated with BBB disruption in this sample, which is in line with previous negative findings in ApoE4 positive individuals [32,39].

In this study, ageing was only negatively correlated with serum albumin levels and not with Qalb, but the age range was small (60–80 years) when compared to other studies that have investigated the effect of ageing on Qalb across the lifespan, so in our data the variability might not be enough to detect those differences. On the other hand, we were able to confirm that there are sex differences in Qalb, which is in line with previous data showing males as having higher Qalb values, and therefore higher BBB dysfunction [35]. The correlation between MMSE and lower levels of Aβ42 has previously been reported [47], whilst in line with previous findings, Qalb was not associated with clinical progression as measured by MMSE or the biochemical transition between AD pathologic change to AD [39]. In previous studies, other associations with age of onset have either found no difference [30] or an increased IgG synthesis in late-onset AD patients [48]. Some evidence shows that Qalb might not be associated with hippocampal atrophy [32], but can instead reflect overall medial temporal lobe atrophy [31], which has also been shown using the IgG index [49]. Another study found a significant negative correlation between the increase in Qalb and 18-Fludeoxyglucose (FDG-PET) uptake in Brodmann Area 42 and 22, which lie within the left superior temporal gyrus, with higher Qalb values being related to a reduced glucose consumption in these areas [50]. In the same study, no significant correlation was observed between brain glucose consumption and IgG index [50]. Similarly, another study showed no correlation between amyloid-PET load and Qalb [39].

Our work has several limitations. Firstly, all participants had perceived memory complaints and objective low memory scores at testing, and data on healthy controls without cognitive impairment were not acquired. This hugely hinders the interpretation of the results since a physiological baseline would be advisable. Nevertheless, we were able to compare patients with memory impairment within the ATN framework, with a cohort of ATN negative individuals, and proved that Qalb impairment is independent from their ATN profile. Secondly, the numbers in each ATN subtype were relatively small (from *n* = 21 to *n* = 70), and a bigger sample might be needed to confirm these findings. Moreover, other biological measures acquired for research purposes such as brain MRI, FDG, amyloid or tau PET, and blood-based biomarkers of AD were not available for this dataset.

## 4. Materials and Methods

Participants: A total of 172 patients with memory impairment were recruited from the Memory Clinic of the Tor Vergata General Hospital of Rome between 2014 and 2017. All patients underwent a comprehensive clinical investigation, including medical history, neurological examination, blood screening for non-neurodegenerative causes of dementia, neuropsychological assessment (MMSE), magnetic resonance imaging (MRI), and CSF analysis. Exclusion criteria were the presence of a major psychiatric disorder, autoimmune, paraneoplastic and infectious disorders, chronic or acute polyneuropathy, radiological evidence of recent ischemic lesions, or any know condition that could alter CSF results due to the CSF sampling procedure such as lumbar spinal stenosis or malignancies.

CSF and Serum Biomarker Analysis: CSF collection was performed in the morning on fasting patients. The first 12 mL of CSF were collected in a polypropylene tube and directly transported to the local laboratory for centrifugation (2000× *g* at +4 °C) for 10 min. The supernatant was pipetted off, mixed to avoid potential gradient effects, aliquoted in 1 mL portions in polypropylene tubes, and stored at −80 °C pending biochemical analyses. The level of CSF biomarkers Aβ40, Aβ42, t-tau, and p-tau phosphorylated at Thr-181 were determined using a sandwich enzyme-linked immunosorbent assay (Fujirebio). Genotyping for ApoE was performed using real-time PCR on the LightCycler instrument (Roche) [51]. Blood-contaminated CSF samples were excluded to avoid false positive values of CSF albumin and protein. Total protein, albumin, and IgG were measured with standard immunochemical nephelometry in CSF and serum using a polyclonal antibody in the case of albumin and IgG. Cut-offs for ATN status were A+: Aβ42/40 ratio < 0.06, T+: p-tau > 60 pg/mL, and N+: t-tau > 350 pg/mL. Concentrations of IgG and albumin in serum and CSF were measured by means of a nephelometer BN ProSpec (Siemens Healthcare Diagnostics, Marburg, Germany). The Qalb was calculated using the formula (CSF Albumin/Serum Albumin) × 1000. IgG index was calculated as follows: [(CSF IgG/serum IgG) × (Serum albumin/CSF albumin)]. Normal BBB permeability was defined as a Qalb < 6.5 if age <40 years, and Qalb < 8.0 if age >40 years as per standard practice [52].

Statistical analysis: Statistical analysis was conducted with MATLAB (ver R2019a) and JASP (JASP team, 2019). According to the normality of the data, between-group differences within the ATN framework were analysed using either a frequentist one-way analysis of variance (ANOVA) or Kruskal–Wallis H test, followed by Bonferroni post-hoc correction. Based on ApoE genotype, a value was assigned according to the graded risk factor associated to each genotype, with consecutive values given to E2/E2, E2/E3, E3/E3, E2/E4, E3/E4, and E4/E4 according to Reiman et al. [53]. Sex correlations are calculated as male/female ratio, with negative values implying an increased male/female ratio. Correlations between continuous variables were investigated using linear regression. Principal component analysis (PCA) using an oblique promax rotation was used to reduce data dimensionality among multiple variables. Statistical significance was set at *p*-value < 0.05.

## 5. Conclusions

Our data showed that BBB dysfunction does not change across different ATN subtypes and that is not correlated with cognitive impairment. However, BBB damage, if measured by Qalb, is associated with low Aβ40 levels, which might potentially have implications for the fine-grain balance between different amyloid species. BBB disruption was not modulated by ApoE status, which, however, showed a dose-dependent effect on the Aβ42/40 ratio. Further studies are needed to replicate these findings including a cohort of cognitively unimpaired individuals and to investigate how blood-based AD-specific biomarkers are impacted by a disruption of the BBB. We hope that this data will highlight the need for further research in this field, which might be crucial for diagnostic and therapeutic purposes.

## Figures and Tables

**Table 1:** Demographics and CSF AD biomarkers within the ATN framework.

	Normal ATN (*n* = 57)	Alzheimer’s Pathologic Change (A+T-N-) (*n* = 21)	Alzheimer’s Disease (A+T+N-, A+T+N+) (*n* = 70)	Non-Alzheimer’s Pathologic Change (A-T+N-, A-T+N+) (*n* = 24)	*p*-Value
Age, years	68.3 ± 8.7	69.48 ± 7.9	71.9 ± 6.7	70.5 ± 8.2	0.085
Sex (M/F ratio)	0.49	0.29	0.51	0.41	0.290
MMSE	23.71 ± 4.7	24.5 ± 5.7	22.8 ± 5.2	25.1 ± 3.2	0.397
ApoE status	2.3	2.5	2.7	2.3	0.285
CSF total-tau	198.6 ± 65.1	247.8 ± 65.8	702.8 ± 397.0	511.0 ± 383.7	<0.001
CSF p-tau	28.1 ± 8.7	37.7 ± 10.2	85.6 ± 36.8	59.7 ± 16.1	<0.001
CSF Aβ42	656.9 ± 223.3	519.2 ± 218.3	498.9 ± 197.4	872.6 ± 255.3	<0.001
CSF Aβ40	7287 ± 2863	10,280 ± 4148	13,270 ± 4312	11,694 ± 3937	<0.001
Aβ42/ 40 ratio	0.09 ± 0.02	0.051 ± 0.01	0.039 ± 0.01	0.077 ± 0.02	<0.001

Values shown as (mean ± SD). Concentrations expressed as pg/mL.

**Table 2:** BBB biomarkers within the ATN framework.

	Normal ATN (*n* = 57)	Alzheimer’s Disease Pathologic Change (*n* = 21)	Alzheimer’s Disease (*n* = 70)	Non-Alzheimer’s Pathologic Change (*n* = 24)	*p*-Value
CSF Protein	51.1 ± 23.6	47.7 ± 12.1	48.9 ± 16.3	49.5 ± 26.1	0.085
Serum Albumin	4103 ± 448	4030 ± 642	4042 ± 443	4204 ± 360	0.897
CSF Albumin	29.5 ± 14.3	28.2 ± 9.5	28.0 ± 12.8	31.9 ± 18.5	0.465
Qalb	7.6 ± 4.4	7.2 ± 3.0	6.9 ± 3.0	7.6 ± 4.6	0.690
Serum IgG	944.7 ± 234	970.3 ± 256	960.0 ± 236	931.3 ± 196	0.931
CSF IgG	3.7 ± 3.1	3.2 ± 1.3	3.1 ± 1.4	3.4 ± 1.5	0.454
IgG Index	0.50 ± 0.1	0.48 ± 0.1	0.47 ± 0.0	0.49 ± 0.1	0.106

**Table 3:** PCA factor loading.

	RC1	RC2	RC3	RC4	RC5
T-tau		0.832			
P-tau		0.926			
Aβ40		0.813	0.448		
Aβ42			0.851		
Aβ42/40 ratio		−0.768			
MMSE			0.497		
ApoE status			−0.564		
Sex	−0.499				
Age				−0.701	
Serum Albumin				0.781	
Serum IgG					0.889
CSF Albumin	0.936				
CSF IgG	0.836				
CSF Protein	0.899				
IgG Index					−0.528
Qalb	0.944				

Only factor loadings >0.4 are presented.

**Table 4:** PCA component correlations.

	RC1	RC2	RC3	RC4	RC5
RC1	1	−0.751		−0.919	0.483
RC2	−0.751	1		0.680	
RC3			1		
RC4	−0.919	0.680		1	−0.470
RC5	0.483			−0.470	1

## Data Availability

The dataset is available upon reasonable request.

## Supplementary Materials

The supporting information can be downloaded at: https://www.mdpi.com/article/10.3390/ijms241512151/s1.
