# Supplementary material for: Blood–Brain Barrier Dysfunction and Aβ42/40 Ratio Dose-Dependent Modulation with the ApoE Genotype within the ATN Framework
Source: Int J Mol Sci. 2023 Jul 29;24(15):12151. doi: 10.3390/ijms241512151 (PMC10418506; doi:10.3390/ijms241512151)
Supplement: Supplementary file 1 [file ijms-24-12151-s001.zip › ijms-2469192-supplementary.pdf]

Supplementary materials

Table S1. S. Post-hoc corrections of group comparisons**.**

	Normal/APC	Normal/AD	Normal/NAPC	APC/AD	APC/NAPC	AD/NAPC
**CSF total-tau**	1	< 0.001	< 0.001	< 0.001	0.019	0.040
**CSF p-tau**	0.826	< 0.001	< 0.001	< 0.001	0.023	<0.001
**CSF Aβ42**	0.084	< 0.001	< 0.001	1	< 0.001	<0.001
**CSF Aβ40**	0.009	< 0.001	< 0.001	0.016	1	0.706
**Aβ42/40 ratio**	< 0.001	< 0.001	< 0.001	0.010	< 0.001	<0.001

Normal = normal ATN profile, APC = Alzheimer's pathologic change, AD = Alzheimer's Disease, NAPC = Non-Alzheimer's pathologic change.
